# (*E*)-5-[3-Cyano-2-(dicyano­methyl­ene)-1-oxaspiro­[4.5]dec-3-en-4-yl]-3-(1-methyl-1,4-dihydro­pyridin-4-yl­idene)pent-4-en-1-yl 3,5-bis­(benz­yloxy)benzoate

**DOI:** 10.1107/S1600536812050532

**Published:** 2012-12-19

**Authors:** Graeme J. Gainsford, David J. Clarke, Andrew J. Kay

**Affiliations:** a Carbohydrate Chemistry Group, Industrial Research Limited, PO Box 31-310, Lower Hutt, New Zealand; b Photonics Group, Industrial Research Limited, PO Box 31-310, Lower Hutt, New Zealand 5040

## Abstract

In the title compound, C_45_H_40_N_4_O_5_, the cyclo­hexane entity on the (3-cyano-2,5-dihydro­furan-2-yl­idene)propane­dinitrile group, which replaces the usual dimethyl substituents, has not perturbed the delocalization geometry significantly. Weak inter­molecular inter­actions, *viz.* C—H⋯N(cyano), C—H⋯O(ether), C—H⋯π and π–π [between the aromatic rings with the shortest centroid–centroid distance of 3.603 (3) Å], consolidate the crystal packing, which exhibits voids of 57 Å^3^.

## Related literature

For related structures, see Bhuiyan *et al.* (2011 ▶); Gainsford *et al.* (2008 ▶, 2013 ▶); Gainsford, Anderson *et al.* (2011 ▶); Gainsford, Ashraf & Kay (2011 ▶). For hydrogen-bonding motifs, see: Bernstein *et al.* (1995 ▶). For calculation software, see: Marder *et al.* (1993 ▶).
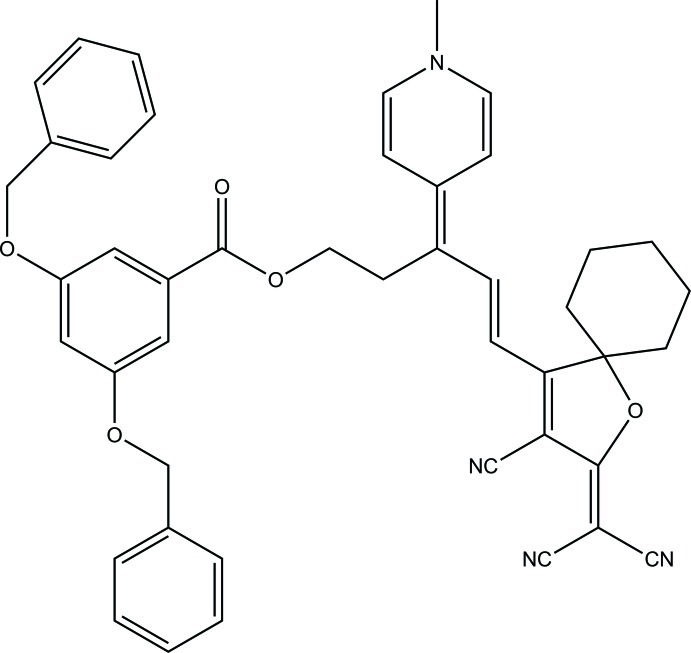


## Experimental

### 

#### Crystal data

C_45_H_40_N_4_O_5_
*M* *_r_* = 716.81
Monoclinic, 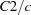

*a* = 29.7208 (16) Å
*b* = 16.0089 (4) Å
*c* = 16.5105 (6) Å
β = 106.705 (5)°
*V* = 7524.1 (5) Å^3^
*Z* = 8
Cu *K*α radiation
μ = 0.67 mm^−1^
*T* = 120 K
0.49 × 0.45 × 0.01 mm

#### Data collection

Oxford Diffraction SuperNova (Dual, Cu at zero, Atlas) diffractometer
Absorption correction: multi-scan (*CrysAlis PRO*; Oxford Diffraction, 2007 ▶) *T*_min_ = 0.638, *T*_max_ = 1.000
31832 measured reflections
6878 independent reflections
4748 reflections with *I* > 2σ(*I*)
*R*_int_ = 0.071

#### Refinement

*R*[*F* ^2^ > 2σ(*F* ^2^)] = 0.048
*wR*(*F* ^2^) = 0.144
*S* = 1.02
6878 reflections
488 parameters
H-atom parameters constrained
Δρ_max_ = 0.26 e Å^−3^
Δρ_min_ = −0.23 e Å^−3^

### 

Data collection: *CrysAlis PRO* (Oxford Diffraction, 2007 ▶); cell refinement: *CrysAlis PRO*; data reduction: *CrysAlis PRO*; program(s) used to solve structure: *SHELXS97* (Sheldrick, 2008 ▶); program(s) used to refine structure: *SHELXL97* (Sheldrick, 2008 ▶); molecular graphics: *ORTEP* in *WinGX* (Farrugia, 2012) ▶ and *Mercury* (Macrae *et al.*, 2008 ▶); software used to prepare material for publication: *SHELXL97* and *PLATON* (Spek, 2009 ▶).

## Supplementary Material

Crystal structure: contains datablock(s) global, I. DOI: 10.1107/S1600536812050532/cv5370sup1.cif

Structure factors: contains datablock(s) I. DOI: 10.1107/S1600536812050532/cv5370Isup2.hkl

Supplementary material file. DOI: 10.1107/S1600536812050532/cv5370Isup3.cml

Additional supplementary materials:  crystallographic information; 3D view; checkCIF report

## Figures and Tables

**Table 1:** Hydrogen-bond geometry (Å, °) *Cg*1 is the centroid of the C33–C38 phenyl ring.

*D*—H⋯*A*	*D*—H	H⋯*A*	*D*⋯*A*	*D*—H⋯*A*
C8—H8*A*⋯N3^i^	0.99	2.54	3.504 (3)	163
C19—H19⋯N2^ii^	0.95	2.47	3.347 (3)	153
C22—H22*B*⋯N2^ii^	0.98	2.59	3.454 (3)	147
C29—H29⋯O5^iii^	0.95	2.54	3.430 (3)	156
C12—H12*A*⋯*Cg*1^iii^	0.99	2.55	3.542 (3)	177

Symmetry codes: (i) 
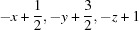
; (ii) 
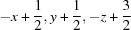
; (iii) 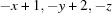.

**Fig. 1.**
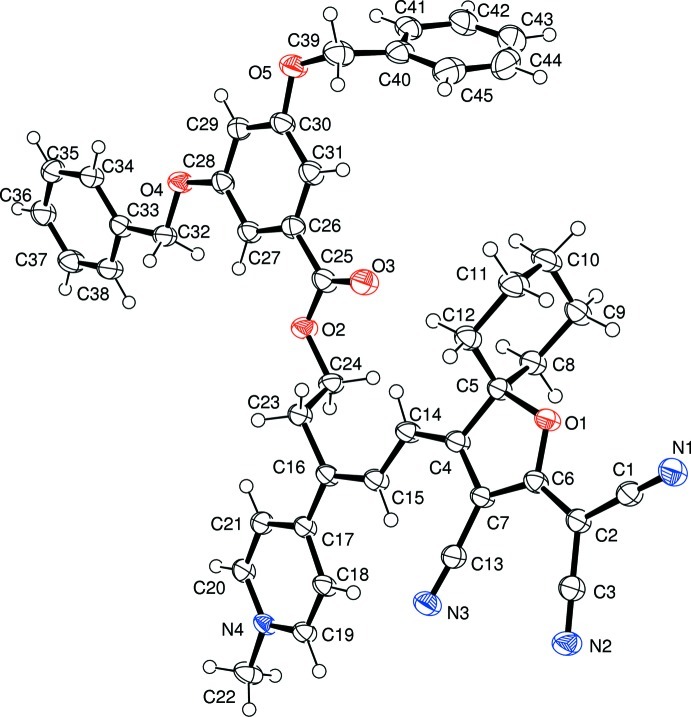
ORTEP (Farrugia, 1999) view of (I) showing the atomic numbering and 30% probability displacement ellipsoids.

**Fig. 2.**
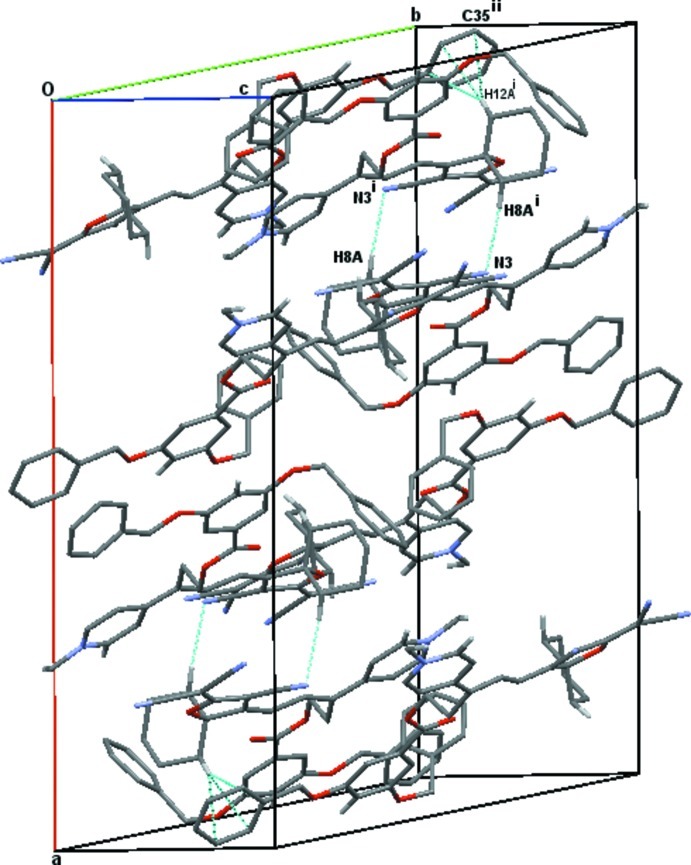
A portion of the crystal packing showing intermolecular binding contacts (R22(14) and C–H···π) as blue dotted lines [symmetry codes: (i) 1/2 - x, 3/2 - y, 1 - z; (ii) 1/2 - x, y - 1/2, 1/2 - z].

## supplementary crystallographic information

### Comment

The title compound, (I), was synthesized as part of our ongoing research involving the development of organic nonlinear optical (NLO) chromophores. We have previously reported the crystallographic parameters for chromophores containing the 2-(3-cyano-4,5,5-trimethyl-5*H*-furan-2-ylidene)-malononitrile electron acceptor group (Bhuiyan *et al.*, 2011). It was hoped that replacement of the 5,5 dimethyl substituents with a fused ring cylcohexane system would retain the donor-acceptor attributes whilst opening up more appropriate molecular packing arrangements for an improved NLO response.

In (I) (Fig. 1), the 5-membered ring plane of atoms O1,C4—C7 (hereafter "CCFP", [3-cyano-5,5-cyclohexane-2,5-dihydrofuran-2-ylidene]propanedinitrile) can be regarded as planar with maximum deviations for C4 & C5 of 0.010 (2) Å. The dicyano group (N1,C1,C2,C3,N2,C6) is planar but twisted by 5.04 (15)° with respect to the "CCFP" group; this is similar to the twist of 5.69 (17)° in related compound 2-{3-cyano-4-[3-(1-decyl-1,4-dihydroquinolin-4-ylidene)prop-1-enyl]- 5,5-dimethyl-2,5-dihydrofuran-2-ylidene}malononitrile (Gainsford *et al.*, 2008), consistent with alleviating intramolecular contacts with the cyano group (C10–N3) and unaffected by the C5,C8–C12 ring atoms. The dihydropyridin-3-yl ring is planar and forms a dihedral angle of 16.33 (13)° with the "CCFP" mean plane. The plane of the connecting polyene atoms (C14–C16) form angles of 10.5 (3)° and 8.4 (2)° with the "CCFP" and dihydropyridin-3-yl ring, respectively, indicating a progressive twist along the molecule.

The remaining atoms of the molecule, excluding one of the terminal benzoyloxy ring atoms C39–C45 and the cyclohexane ring atoms C8—C12, are almost in parallel planes with dihedral angles for the C26–C31 and C33–C38 ring planes to the dihydropyridin-3-yl being 4.89 (11)° and 17.25 (11)°, respectively. The benzyloxy ring C30–C45 is twisted away from its parent benzene ring (C26–C31) with an interplanar dihedral angle of 76.24 (13)°. The benzoyloxy ring C33—C38 is constrained to its near coplanar position by intermolecular C—H···O and C–H···π interactions (Table 1). Overall the affect of the dimethyl replacement with a cyclohexane moiety has not changed the observed delocalized geometry with a BLA value of -0.041 Å (Marder *et al.*, 1993) compared with 2-[4-(2-{5-*tert*-Butyl-2-chloro-3-[2-(3-pentyl-3*H*-benzothiazol-2-ylidene) -ethylidene]-cyclohex-1-enyl}-vinyl)-3-cyano-5,5-dimethyl-5*H*-furan -2-ylidene]-malononitrile (Gainsford *et al.*, 2013) of -0.026 Å.

As was frequently observed for such compounds (Gainsford *et al.*, 2013), the molecules pack into dimeric units about centres of symmetry utilizing in this case multiple *C*–*H*···cyano(N), one C–H···O, and one C–H···π attractive interactions. Ring *R*^2^_2_(14) & *R*^2^_2_(8) motifs (Bernstein *et al.*, 1995) about the centres are observed with additional chain C(13) & C(15) motifs. Table 1 summarizes the most attractive interactions and key elements are shown in Figure 2; there are several weaker C–H···N(cyano) close contacts not listed. The cyano atom N2 forms bifurcated donor interactions generating a *R*^1^_2_(6) motif.

### Experimental

In a conical flask fitted with a reflux condenser were added *N*-(2-(3-cyano-2-(dicyanomethylene)-1-oxaspiro[4.5]dec-3-en-4-yl)vinyl) -*N*-phenylacetamide (5 mmol), 4-(3-((3,5-bis(benzyloxy)benzoyl)oxy)propyl)-1-methylpyridin-1-ium iodide (5 mmol), triethylamine (5 mmol) and acetic anhydride (40 ml). The mixture was stirred at 80 °C overnight and then left to cool to room temperature. The solvent was removed and the product precipitated with the addition of diethyl ether (100 mL). The precipitate was collected by filtration and washed with diethyl ether (3 *x* 50 ml) and dried under vacuum. The resultant product was purified by column chromatography (ethyl acetate/acetone 4:1).

ESI 716.2992 (exptl) 716.2999 (calc) for C_45_H_40_N_4_O_5_ (Δ 1.0ppm). ^1^H NMR (500 MHz, d_6_-DMSO); 8.52 (d, *J* 7.2, rotamer 1 PyH, 1.8H), 8.45 (d, *J* 7.2, rotamer 2 PyH, 0.2H), 8.38 (d, *J* 12.5, rotamer 1 CH, 0.9H), 7.90 (d, *J* 7.1, rotamer 2, PyH, 0.2H), 7.75 (d, *J* 7.2, rotamer 1 PyH, 1.8H), 7.51 (d, *J* 13.0, rotamer 2 CH, 0.1H), 7.44–7.39 (m, ArH, 8H), 7.36–7.32 (m, ArH, 2H), 7.04–7.03 (m, ArH, 2H), 6.95 (t, *J* 2.3, rotamer 1 ArH, 0.9H), 6.92 (t, *J* 2.3, rotamer 2 ArH, 0.1H), 5.10 (s, rotamer 1 Bz CH2, 3.6H), 5.09 (s, rotamer 2 Bz CH2, 0.4H), 4.35 (t, *J* 6.4, CH2, 2H), 4.03 (s, NCH3, 3H), 3.09 (t, *J* 6.4, CH2, 2H), 1.71–1.42 (m, Cy CH2, 8H), 1.31–1.23 (m, Cy CH2, 8H). ^13^C NMR (125 MHz, d_6_-DMSO); 176.2, 165.4, 159.5, 159.4, 154.7, 143.5, 136.5, 136.0, 131.6, 128.5, 128.0, 127.7, 120.5, 120.1, 119.6, 118.6, 117.3, 116.5, 108.1, 106.4, 100.9, 93.8, 71.1, 69.6, 62.9, 45.6, 35.2, 25.2, 23.7, 21.6

### Refinement

Twenty-one outlier reflection identified by large Δ/σ(F^2^) ratios (>3.5) including two affected by backstop scatter were OMITted from the dataset. All methyl H atoms were constrained to an ideal geometry (C—H = 0.98 Å) with *U*_iso_(H) = 1.5*U*_eq_(C), but were allowed to rotate freely about the adjacent C—C bond. All other C bound H atoms were placed in geometrically idealized positions and constrained to ride on their parent atoms with C—H distances of 1.00 (primary), 0.99 (methylene) or 0.95 (phenyl) Å and with *U*_iso_(H) = 1.2*U*_eq_(C).

### Crystal data

**Table:**

C_45_H_40_N_4_O_5_	*F*(000) = 3024
*M**_r_* = 716.81	*D*_x_ = 1.266 Mg m^−^^3^
Monoclinic, *C*2/*c*	Cu *K*α radiation, λ = 1.54184 Å
Hall symbol: -C 2yc	Cell parameters from 5455 reflections
*a* = 29.7208 (16) Å	θ = 2.8–73.8°
*b* = 16.0089 (4) Å	µ = 0.67 mm^−^^1^
*c* = 16.5105 (6) Å	*T* = 120 K
β = 106.705 (5)°	Plate, yellow
*V* = 7524.1 (5) Å^3^	0.49 × 0.45 × 0.01 mm
*Z* = 8	

### Data collection

**Table:**

Oxford Diffraction SuperNova (Dual, Cu at zero, Atlas) diffractometer	6878 independent reflections
Radiation source: SuperNova (Cu) X-ray Source	4748 reflections with *I* > 2σ(*I*)
Mirror monochromator	*R*_int_ = 0.071
Detector resolution: 10.6501 pixels mm^-1^	θ_max_ = 68.2°, θ_min_ = 3.2°
ω scans	*h* = −35→35
Absorption correction: multi-scan (*CrysAlis PRO*; Oxford Diffraction, 2007)	*k* = −19→19
*T*_min_ = 0.638, *T*_max_ = 1.000	*l* = −19→17
31832 measured reflections	

### Refinement

**Table:**

Refinement on *F*^2^	Primary atom site location: structure-invariant direct methods
Least-squares matrix: full	Secondary atom site location: difference Fourier map
*R*[*F*^2^ > 2σ(*F*^2^)] = 0.048	Hydrogen site location: inferred from neighbouring sites
*wR*(*F*^2^) = 0.144	H-atom parameters constrained
*S* = 1.02	*w* = 1/[σ^2^(*F*_o_^2^) + (0.0691*P*)^2^ + 2.9516*P*] where *P* = (*F*_o_^2^ + 2*F*_c_^2^)/3
6878 reflections	(Δ/σ)_max_ < 0.001
488 parameters	Δρ_max_ = 0.26 e Å^−^^3^
0 restraints	Δρ_min_ = −0.23 e Å^−^^3^

### Special details

**Table:**

Experimental. Empirical absorption correction using spherical harmonics, implemented in *CrysAlis PRO* (Oxford Diffraction, 2007)
Geometry. All e.s.d.'s (except the e.s.d. in the dihedral angle between two l.s. planes) are estimated using the full covariance matrix. The cell e.s.d.'s are taken into account individually in the estimation of e.s.d.'s in distances, angles and torsion angles; correlations between e.s.d.'s in cell parameters are only used when they are defined by crystal symmetry. An approximate (isotropic) treatment of cell e.s.d.'s is used for estimating e.s.d.'s involving l.s. planes.
Refinement. Refinement of *F*^2^ against ALL reflections. The weighted *R*-factor *wR* and goodness of fit *S* are based on *F*^2^, conventional *R*-factors *R* are based on *F*, with *F* set to zero for negative *F*^2^. The threshold expression of *F*^2^ > σ(*F*^2^) is used only for calculating *R*-factors(gt) *etc*. and is not relevant to the choice of reflections for refinement. *R*-factors based on *F*^2^ are statistically about twice as large as those based on *F*, and *R*- factors based on ALL data will be even larger.

### Fractional atomic coordinates and isotropic or equivalent isotropic displacement parameters (Å^2^)

**Table:**

	*x*	*y*	*z*	*U*_iso_*/*U*_eq_	
O1	0.32530 (6)	0.58411 (8)	0.47050 (9)	0.0302 (3)	
O2	0.37640 (6)	1.01270 (9)	0.29493 (10)	0.0349 (4)	
O3	0.38897 (6)	0.87595 (9)	0.27559 (11)	0.0418 (4)	
O4	0.46422 (6)	1.17572 (9)	0.13475 (10)	0.0353 (4)	
O5	0.49344 (6)	0.90251 (10)	0.07098 (11)	0.0403 (4)	
N1	0.29766 (7)	0.39441 (11)	0.55045 (13)	0.0371 (5)	
N2	0.26282 (8)	0.59452 (11)	0.70530 (13)	0.0403 (5)	
N3	0.30967 (7)	0.78188 (11)	0.67582 (12)	0.0361 (4)	
N4	0.28722 (6)	1.15634 (10)	0.61141 (11)	0.0279 (4)	
C1	0.29686 (8)	0.46514 (13)	0.56464 (14)	0.0299 (5)	
C2	0.29536 (8)	0.55113 (12)	0.58331 (14)	0.0284 (5)	
C3	0.27750 (8)	0.57533 (12)	0.65055 (14)	0.0307 (5)	
C4	0.33533 (8)	0.73015 (12)	0.48301 (14)	0.0281 (5)	
C5	0.34064 (8)	0.65630 (12)	0.42888 (13)	0.0278 (4)	
C6	0.31187 (7)	0.61102 (12)	0.53672 (13)	0.0262 (4)	
C7	0.31686 (7)	0.69802 (12)	0.54721 (13)	0.0263 (4)	
C8	0.30667 (8)	0.66319 (13)	0.34007 (14)	0.0320 (5)	
H8A	0.2742	0.6687	0.3442	0.038*	
H8B	0.3140	0.7142	0.3124	0.038*	
C9	0.30951 (9)	0.58736 (14)	0.28600 (15)	0.0356 (5)	
H9A	0.2986	0.5372	0.3100	0.043*	
H9B	0.2885	0.5958	0.2280	0.043*	
C10	0.35947 (9)	0.57340 (15)	0.28232 (16)	0.0409 (6)	
H10A	0.3692	0.6210	0.2530	0.049*	
H10B	0.3606	0.5221	0.2495	0.049*	
C11	0.39340 (9)	0.56480 (14)	0.37067 (16)	0.0391 (6)	
H11A	0.4258	0.5599	0.3664	0.047*	
H11B	0.3860	0.5131	0.3972	0.047*	
C12	0.39053 (8)	0.63983 (13)	0.42687 (15)	0.0320 (5)	
H12A	0.4106	0.6292	0.4851	0.038*	
H12B	0.4028	0.6901	0.4054	0.038*	
C13	0.31180 (8)	0.74285 (12)	0.61819 (14)	0.0285 (5)	
C14	0.34625 (8)	0.81009 (12)	0.46547 (14)	0.0296 (5)	
H14	0.3625	0.8181	0.4242	0.035*	
C15	0.33433 (8)	0.88138 (12)	0.50617 (14)	0.0286 (5)	
H15	0.3167	0.8713	0.5449	0.034*	
C16	0.34535 (8)	0.96279 (12)	0.49610 (13)	0.0266 (4)	
C17	0.32635 (7)	1.02850 (12)	0.53696 (13)	0.0270 (4)	
C18	0.30183 (8)	1.01255 (12)	0.59721 (13)	0.0291 (5)	
H18	0.2986	0.9566	0.6137	0.035*	
C19	0.28272 (8)	1.07569 (12)	0.63225 (14)	0.0298 (5)	
H19	0.2660	1.0627	0.6718	0.036*	
C20	0.31179 (8)	1.17533 (12)	0.55675 (14)	0.0305 (5)	
H20	0.3156	1.2322	0.5437	0.037*	
C21	0.33127 (8)	1.11403 (13)	0.51969 (14)	0.0303 (5)	
H21	0.3485	1.1293	0.4816	0.036*	
C22	0.26343 (9)	1.22221 (13)	0.64581 (16)	0.0382 (5)	
H22A	0.2808	1.2747	0.6493	0.057*	
H22B	0.2622	1.2061	0.7024	0.057*	
H22C	0.2314	1.2296	0.6087	0.057*	
C23	0.37562 (8)	0.98383 (12)	0.43915 (13)	0.0300 (5)	
H23A	0.3923	1.0371	0.4579	0.036*	
H23B	0.3995	0.9396	0.4441	0.036*	
C24	0.34667 (8)	0.99168 (14)	0.34785 (14)	0.0332 (5)	
H24A	0.3303	0.9382	0.3286	0.040*	
H24B	0.3226	1.0356	0.3427	0.040*	
C25	0.39534 (8)	0.94887 (13)	0.26244 (14)	0.0330 (5)	
C26	0.42416 (8)	0.98005 (13)	0.20857 (13)	0.0305 (5)	
C27	0.42961 (8)	1.06554 (13)	0.19995 (13)	0.0302 (5)	
H27	0.4156	1.1043	0.2291	0.036*	
C28	0.45591 (8)	1.09321 (13)	0.14781 (14)	0.0307 (5)	
C29	0.47602 (8)	1.03630 (14)	0.10522 (14)	0.0326 (5)	
H29	0.4936	1.0556	0.0691	0.039*	
C30	0.47045 (8)	0.95139 (14)	0.11528 (14)	0.0323 (5)	
C31	0.44396 (8)	0.92181 (14)	0.16634 (14)	0.0325 (5)	
H31	0.4395	0.8636	0.1722	0.039*	
C32	0.44280 (8)	1.23607 (13)	0.17648 (14)	0.0315 (5)	
H32A	0.4547	1.2287	0.2385	0.038*	
H32B	0.4083	1.2282	0.1592	0.038*	
C33	0.45466 (8)	1.32258 (13)	0.15247 (14)	0.0307 (5)	
C34	0.49068 (8)	1.33723 (14)	0.11593 (14)	0.0318 (5)	
H34	0.5086	1.2918	0.1050	0.038*	
C35	0.50046 (8)	1.41794 (14)	0.09546 (15)	0.0359 (5)	
H35	0.5250	1.4275	0.0703	0.043*	
C36	0.47465 (9)	1.48459 (14)	0.11152 (16)	0.0409 (6)	
H36	0.4814	1.5398	0.0974	0.049*	
C37	0.43883 (9)	1.47050 (15)	0.14829 (17)	0.0421 (6)	
H37	0.4210	1.5162	0.1591	0.050*	
C38	0.42898 (9)	1.38999 (14)	0.16940 (15)	0.0370 (5)	
H38	0.4048	1.3808	0.1954	0.044*	
C39	0.49099 (9)	0.81346 (14)	0.07664 (17)	0.0425 (6)	
H39A	0.4923	0.7980	0.1354	0.051*	
H39B	0.5187	0.7887	0.0640	0.051*	
C40	0.44692 (9)	0.77678 (14)	0.01700 (17)	0.0395 (6)	
C41	0.42830 (9)	0.80771 (14)	−0.06413 (16)	0.0393 (6)	
H41	0.4419	0.8560	−0.0811	0.047*	
C42	0.39010 (10)	0.76945 (17)	−0.1214 (2)	0.0519 (7)	
H42	0.3773	0.7921	−0.1764	0.062*	
C43	0.37105 (11)	0.6981 (2)	−0.0972 (3)	0.0637 (9)	
H43	0.3456	0.6705	−0.1363	0.076*	
C44	0.38874 (12)	0.66699 (18)	−0.0166 (3)	0.0687 (10)	
H44	0.3751	0.6186	−0.0001	0.082*	
C45	0.42647 (11)	0.70607 (16)	0.0409 (2)	0.0531 (7)	
H45	0.4383	0.6845	0.0965	0.064*	

### Atomic displacement parameters (Å^2^)

**Table:**

	*U*^11^	*U*^22^	*U*^33^	*U*^12^	*U*^13^	*U*^23^
O1	0.0500 (9)	0.0187 (7)	0.0275 (8)	−0.0009 (6)	0.0203 (7)	0.0004 (6)
O2	0.0514 (9)	0.0287 (8)	0.0315 (9)	−0.0031 (7)	0.0229 (7)	0.0020 (6)
O3	0.0637 (11)	0.0274 (8)	0.0424 (10)	−0.0032 (7)	0.0283 (9)	0.0026 (7)
O4	0.0502 (9)	0.0256 (7)	0.0385 (9)	−0.0032 (6)	0.0259 (8)	−0.0019 (6)
O5	0.0529 (10)	0.0316 (8)	0.0441 (10)	−0.0001 (7)	0.0264 (8)	−0.0059 (7)
N1	0.0566 (12)	0.0232 (9)	0.0357 (11)	−0.0008 (8)	0.0200 (10)	0.0020 (8)
N2	0.0648 (14)	0.0276 (9)	0.0385 (12)	−0.0049 (9)	0.0311 (11)	0.0001 (8)
N3	0.0533 (12)	0.0273 (9)	0.0324 (11)	−0.0017 (8)	0.0200 (9)	−0.0034 (8)
N4	0.0425 (10)	0.0193 (8)	0.0235 (9)	−0.0008 (7)	0.0119 (8)	−0.0022 (7)
C1	0.0402 (12)	0.0266 (11)	0.0248 (11)	−0.0013 (9)	0.0127 (9)	0.0048 (8)
C2	0.0415 (12)	0.0203 (9)	0.0258 (11)	−0.0011 (8)	0.0134 (9)	0.0017 (8)
C3	0.0453 (13)	0.0198 (9)	0.0304 (12)	0.0002 (9)	0.0160 (10)	0.0033 (8)
C4	0.0371 (11)	0.0237 (10)	0.0265 (11)	0.0014 (8)	0.0140 (9)	−0.0007 (8)
C5	0.0441 (12)	0.0180 (9)	0.0254 (11)	−0.0009 (8)	0.0167 (9)	0.0020 (8)
C6	0.0348 (11)	0.0228 (10)	0.0219 (11)	0.0010 (8)	0.0095 (9)	0.0004 (8)
C7	0.0382 (11)	0.0207 (9)	0.0231 (11)	0.0002 (8)	0.0139 (9)	−0.0007 (8)
C8	0.0440 (12)	0.0251 (10)	0.0293 (12)	0.0020 (9)	0.0144 (10)	0.0017 (9)
C9	0.0520 (14)	0.0289 (11)	0.0265 (12)	−0.0011 (9)	0.0122 (10)	−0.0024 (9)
C10	0.0617 (16)	0.0323 (11)	0.0360 (13)	0.0013 (11)	0.0256 (12)	−0.0065 (10)
C11	0.0491 (14)	0.0296 (11)	0.0448 (14)	0.0063 (10)	0.0232 (12)	−0.0022 (10)
C12	0.0420 (12)	0.0257 (10)	0.0311 (12)	0.0017 (9)	0.0148 (10)	−0.0003 (9)
C13	0.0389 (12)	0.0205 (9)	0.0294 (12)	−0.0002 (8)	0.0151 (9)	0.0023 (9)
C14	0.0468 (12)	0.0209 (9)	0.0263 (11)	0.0005 (9)	0.0189 (10)	0.0018 (8)
C15	0.0413 (12)	0.0226 (10)	0.0245 (11)	0.0015 (8)	0.0134 (9)	0.0016 (8)
C16	0.0397 (11)	0.0203 (9)	0.0213 (11)	−0.0012 (8)	0.0111 (9)	0.0001 (8)
C17	0.0389 (11)	0.0208 (9)	0.0227 (11)	−0.0010 (8)	0.0112 (9)	0.0006 (8)
C18	0.0476 (12)	0.0188 (9)	0.0247 (11)	−0.0015 (8)	0.0161 (10)	−0.0009 (8)
C19	0.0459 (12)	0.0232 (10)	0.0236 (11)	−0.0035 (9)	0.0154 (10)	0.0004 (8)
C20	0.0453 (12)	0.0199 (9)	0.0279 (11)	−0.0033 (8)	0.0129 (10)	0.0022 (8)
C21	0.0404 (12)	0.0234 (10)	0.0303 (12)	−0.0034 (8)	0.0152 (10)	0.0006 (9)
C22	0.0617 (15)	0.0226 (10)	0.0361 (13)	0.0051 (10)	0.0233 (12)	−0.0022 (9)
C23	0.0437 (12)	0.0213 (9)	0.0290 (12)	−0.0019 (9)	0.0167 (10)	0.0006 (8)
C24	0.0456 (13)	0.0308 (11)	0.0300 (12)	−0.0032 (9)	0.0216 (10)	0.0007 (9)
C25	0.0453 (13)	0.0288 (11)	0.0261 (11)	−0.0015 (9)	0.0123 (10)	−0.0015 (9)
C26	0.0364 (11)	0.0326 (11)	0.0228 (11)	−0.0025 (9)	0.0088 (9)	0.0009 (9)
C27	0.0393 (12)	0.0294 (10)	0.0240 (11)	−0.0020 (9)	0.0125 (9)	−0.0002 (9)
C28	0.0388 (12)	0.0280 (10)	0.0253 (11)	−0.0038 (9)	0.0095 (9)	−0.0005 (9)
C29	0.0378 (12)	0.0346 (11)	0.0281 (12)	−0.0050 (9)	0.0137 (10)	−0.0025 (9)
C30	0.0385 (12)	0.0349 (11)	0.0247 (12)	−0.0005 (9)	0.0112 (10)	−0.0037 (9)
C31	0.0421 (12)	0.0298 (11)	0.0262 (12)	−0.0012 (9)	0.0106 (10)	−0.0001 (9)
C32	0.0374 (11)	0.0299 (11)	0.0305 (12)	0.0003 (9)	0.0153 (10)	−0.0012 (9)
C33	0.0390 (12)	0.0281 (11)	0.0258 (11)	−0.0002 (9)	0.0105 (9)	−0.0015 (9)
C34	0.0365 (11)	0.0304 (11)	0.0304 (12)	0.0022 (9)	0.0126 (10)	0.0003 (9)
C35	0.0405 (12)	0.0323 (11)	0.0385 (13)	−0.0033 (9)	0.0168 (11)	0.0016 (10)
C36	0.0535 (14)	0.0273 (11)	0.0456 (15)	−0.0036 (10)	0.0200 (12)	−0.0012 (10)
C37	0.0547 (15)	0.0300 (11)	0.0477 (15)	0.0038 (10)	0.0247 (12)	−0.0044 (11)
C38	0.0454 (13)	0.0355 (12)	0.0350 (13)	0.0017 (10)	0.0193 (11)	−0.0019 (10)
C39	0.0541 (15)	0.0309 (12)	0.0472 (15)	0.0084 (10)	0.0223 (12)	0.0016 (11)
C40	0.0494 (14)	0.0252 (11)	0.0531 (16)	0.0061 (9)	0.0293 (12)	−0.0018 (10)
C41	0.0483 (14)	0.0283 (11)	0.0469 (15)	0.0034 (10)	0.0226 (12)	−0.0063 (10)
C42	0.0491 (15)	0.0491 (15)	0.0609 (18)	0.0032 (12)	0.0214 (14)	−0.0192 (13)
C43	0.0525 (17)	0.0538 (17)	0.092 (3)	−0.0068 (14)	0.0328 (18)	−0.0312 (18)
C44	0.073 (2)	0.0346 (14)	0.119 (3)	−0.0119 (14)	0.061 (2)	−0.0152 (17)
C45	0.0688 (18)	0.0306 (12)	0.075 (2)	0.0054 (12)	0.0441 (16)	0.0038 (13)

### Geometric parameters (Å, º)

**Table:**

O1—C6	1.338 (3)	C20—C21	1.370 (3)
O1—C5	1.482 (2)	C20—H20	0.9500
O2—C25	1.350 (3)	C21—H21	0.9500
O2—C24	1.450 (3)	C22—H22A	0.9800
O3—C25	1.212 (3)	C22—H22B	0.9800
O4—C28	1.372 (3)	C22—H22C	0.9800
O4—C32	1.437 (3)	C23—C24	1.511 (3)
O5—C30	1.379 (3)	C23—H23A	0.9900
O5—C39	1.432 (3)	C23—H23B	0.9900
N1—C1	1.158 (3)	C24—H24A	0.9900
N2—C3	1.153 (3)	C24—H24B	0.9900
N3—C13	1.155 (3)	C25—C26	1.487 (3)
N4—C20	1.349 (3)	C26—C27	1.390 (3)
N4—C19	1.353 (3)	C26—C31	1.392 (3)
N4—C22	1.471 (3)	C27—C28	1.391 (3)
C1—C2	1.414 (3)	C27—H27	0.9500
C2—C6	1.403 (3)	C28—C29	1.387 (3)
C2—C3	1.415 (3)	C29—C30	1.385 (3)
C4—C14	1.372 (3)	C29—H29	0.9500
C4—C7	1.423 (3)	C30—C31	1.391 (3)
C4—C5	1.517 (3)	C31—H31	0.9500
C5—C12	1.516 (3)	C32—C33	1.510 (3)
C5—C8	1.527 (3)	C32—H32A	0.9900
C6—C7	1.406 (3)	C32—H32B	0.9900
C7—C13	1.419 (3)	C33—C34	1.392 (3)
C8—C9	1.524 (3)	C33—C38	1.396 (3)
C8—H8A	0.9900	C34—C35	1.387 (3)
C8—H8B	0.9900	C34—H34	0.9500
C9—C10	1.520 (4)	C35—C36	1.383 (3)
C9—H9A	0.9900	C35—H35	0.9500
C9—H9B	0.9900	C36—C37	1.387 (4)
C10—C11	1.522 (4)	C36—H36	0.9500
C10—H10A	0.9900	C37—C38	1.388 (3)
C10—H10B	0.9900	C37—H37	0.9500
C11—C12	1.535 (3)	C38—H38	0.9500
C11—H11A	0.9900	C39—C40	1.513 (4)
C11—H11B	0.9900	C39—H39A	0.9900
C12—H12A	0.9900	C39—H39B	0.9900
C12—H12B	0.9900	C40—C41	1.386 (4)
C14—C15	1.420 (3)	C40—C45	1.394 (3)
C14—H14	0.9500	C41—C42	1.393 (4)
C15—C16	1.365 (3)	C41—H41	0.9500
C15—H15	0.9500	C42—C43	1.384 (4)
C16—C17	1.449 (3)	C42—H42	0.9500
C16—C23	1.514 (3)	C43—C44	1.376 (5)
C17—C21	1.415 (3)	C43—H43	0.9500
C17—C18	1.415 (3)	C44—C45	1.392 (5)
C18—C19	1.367 (3)	C44—H44	0.9500
C18—H18	0.9500	C45—H45	0.9500
C19—H19	0.9500		
			
C6—O1—C5	109.36 (15)	N4—C22—H22C	109.5
C25—O2—C24	117.37 (17)	H22A—C22—H22C	109.5
C28—O4—C32	116.56 (17)	H22B—C22—H22C	109.5
C30—O5—C39	119.21 (19)	C24—C23—C16	111.62 (18)
C20—N4—C19	119.83 (18)	C24—C23—H23A	109.3
C20—N4—C22	120.63 (17)	C16—C23—H23A	109.3
C19—N4—C22	119.48 (18)	C24—C23—H23B	109.3
N1—C1—C2	178.7 (3)	C16—C23—H23B	109.3
C6—C2—C1	120.5 (2)	H23A—C23—H23B	108.0
C6—C2—C3	120.80 (18)	O2—C24—C23	110.47 (18)
C1—C2—C3	118.68 (19)	O2—C24—H24A	109.6
N2—C3—C2	179.5 (3)	C23—C24—H24A	109.6
C14—C4—C7	131.3 (2)	O2—C24—H24B	109.6
C14—C4—C5	122.11 (19)	C23—C24—H24B	109.6
C7—C4—C5	106.55 (17)	H24A—C24—H24B	108.1
O1—C5—C12	107.93 (16)	O3—C25—O2	123.6 (2)
O1—C5—C4	103.68 (16)	O3—C25—C26	125.2 (2)
C12—C5—C4	114.61 (18)	O2—C25—C26	111.17 (18)
O1—C5—C8	107.16 (16)	C27—C26—C31	122.0 (2)
C12—C5—C8	111.88 (18)	C27—C26—C25	119.7 (2)
C4—C5—C8	110.94 (17)	C31—C26—C25	118.28 (19)
O1—C6—C2	117.47 (18)	C26—C27—C28	118.7 (2)
O1—C6—C7	111.94 (18)	C26—C27—H27	120.7
C2—C6—C7	130.6 (2)	C28—C27—H27	120.7
C6—C7—C13	124.77 (19)	O4—C28—C29	115.4 (2)
C6—C7—C4	108.43 (18)	O4—C28—C27	124.2 (2)
C13—C7—C4	126.00 (18)	C29—C28—C27	120.4 (2)
C9—C8—C5	111.87 (18)	C30—C29—C28	120.0 (2)
C9—C8—H8A	109.2	C30—C29—H29	120.0
C5—C8—H8A	109.2	C28—C29—H29	120.0
C9—C8—H8B	109.2	O5—C30—C29	113.5 (2)
C5—C8—H8B	109.2	O5—C30—C31	125.5 (2)
H8A—C8—H8B	107.9	C29—C30—C31	121.0 (2)
C10—C9—C8	111.09 (19)	C30—C31—C26	118.0 (2)
C10—C9—H9A	109.4	C30—C31—H31	121.0
C8—C9—H9A	109.4	C26—C31—H31	121.0
C10—C9—H9B	109.4	O4—C32—C33	108.77 (17)
C8—C9—H9B	109.4	O4—C32—H32A	109.9
H9A—C9—H9B	108.0	C33—C32—H32A	109.9
C9—C10—C11	111.1 (2)	O4—C32—H32B	109.9
C9—C10—H10A	109.4	C33—C32—H32B	109.9
C11—C10—H10A	109.4	H32A—C32—H32B	108.3
C9—C10—H10B	109.4	C34—C33—C38	119.3 (2)
C11—C10—H10B	109.4	C34—C33—C32	122.35 (19)
H10A—C10—H10B	108.0	C38—C33—C32	118.3 (2)
C10—C11—C12	111.83 (19)	C35—C34—C33	120.2 (2)
C10—C11—H11A	109.3	C35—C34—H34	119.9
C12—C11—H11A	109.3	C33—C34—H34	119.9
C10—C11—H11B	109.3	C36—C35—C34	120.3 (2)
C12—C11—H11B	109.3	C36—C35—H35	119.8
H11A—C11—H11B	107.9	C34—C35—H35	119.8
C5—C12—C11	111.96 (18)	C35—C36—C37	119.7 (2)
C5—C12—H12A	109.2	C35—C36—H36	120.1
C11—C12—H12A	109.2	C37—C36—H36	120.1
C5—C12—H12B	109.2	C36—C37—C38	120.3 (2)
C11—C12—H12B	109.2	C36—C37—H37	119.8
H12A—C12—H12B	107.9	C38—C37—H37	119.8
N3—C13—C7	176.4 (2)	C37—C38—C33	120.1 (2)
C4—C14—C15	122.9 (2)	C37—C38—H38	120.0
C4—C14—H14	118.5	C33—C38—H38	120.0
C15—C14—H14	118.5	O5—C39—C40	113.3 (2)
C16—C15—C14	127.5 (2)	O5—C39—H39A	108.9
C16—C15—H15	116.3	C40—C39—H39A	108.9
C14—C15—H15	116.3	O5—C39—H39B	108.9
C15—C16—C17	119.8 (2)	C40—C39—H39B	108.9
C15—C16—C23	119.59 (19)	H39A—C39—H39B	107.7
C17—C16—C23	120.54 (17)	C41—C40—C45	118.6 (3)
C21—C17—C18	114.85 (19)	C41—C40—C39	121.4 (2)
C21—C17—C16	122.15 (19)	C45—C40—C39	119.8 (2)
C18—C17—C16	123.00 (18)	C40—C41—C42	121.3 (2)
C19—C18—C17	121.65 (19)	C40—C41—H41	119.3
C19—C18—H18	119.2	C42—C41—H41	119.3
C17—C18—H18	119.2	C43—C42—C41	119.2 (3)
N4—C19—C18	121.0 (2)	C43—C42—H42	120.4
N4—C19—H19	119.5	C41—C42—H42	120.4
C18—C19—H19	119.5	C44—C43—C42	120.3 (3)
N4—C20—C21	121.15 (19)	C44—C43—H43	119.9
N4—C20—H20	119.4	C42—C43—H43	119.9
C21—C20—H20	119.4	C43—C44—C45	120.4 (3)
C20—C21—C17	121.5 (2)	C43—C44—H44	119.8
C20—C21—H21	119.3	C45—C44—H44	119.8
C17—C21—H21	119.3	C44—C45—C40	120.2 (3)
N4—C22—H22A	109.5	C44—C45—H45	119.9
N4—C22—H22B	109.5	C40—C45—H45	119.9
H22A—C22—H22B	109.5		
			
C6—O1—C5—C12	123.48 (18)	C18—C17—C21—C20	−2.5 (3)
C6—O1—C5—C4	1.5 (2)	C16—C17—C21—C20	177.8 (2)
C6—O1—C5—C8	−115.86 (18)	C15—C16—C23—C24	85.1 (2)
C14—C4—C5—O1	179.8 (2)	C17—C16—C23—C24	−92.2 (2)
C7—C4—C5—O1	−1.7 (2)	C25—O2—C24—C23	89.3 (2)
C14—C4—C5—C12	62.5 (3)	C16—C23—C24—O2	179.38 (16)
C7—C4—C5—C12	−119.1 (2)	C24—O2—C25—O3	−0.4 (3)
C14—C4—C5—C8	−65.4 (3)	C24—O2—C25—C26	179.01 (18)
C7—C4—C5—C8	113.0 (2)	O3—C25—C26—C27	−178.6 (2)
C5—O1—C6—C2	178.95 (18)	O2—C25—C26—C27	2.0 (3)
C5—O1—C6—C7	−0.7 (2)	O3—C25—C26—C31	3.1 (4)
C1—C2—C6—O1	5.3 (3)	O2—C25—C26—C31	−176.36 (19)
C3—C2—C6—O1	−175.6 (2)	C31—C26—C27—C28	−0.4 (3)
C1—C2—C6—C7	−175.1 (2)	C25—C26—C27—C28	−178.7 (2)
C3—C2—C6—C7	4.0 (4)	C32—O4—C28—C29	178.50 (19)
O1—C6—C7—C13	−170.7 (2)	C32—O4—C28—C27	−1.9 (3)
C2—C6—C7—C13	9.6 (4)	C26—C27—C28—O4	−179.3 (2)
O1—C6—C7—C4	−0.4 (3)	C26—C27—C28—C29	0.3 (3)
C2—C6—C7—C4	179.9 (2)	O4—C28—C29—C30	178.8 (2)
C14—C4—C7—C6	179.6 (2)	C27—C28—C29—C30	−0.8 (3)
C5—C4—C7—C6	1.4 (2)	C39—O5—C30—C29	179.3 (2)
C14—C4—C7—C13	−10.3 (4)	C39—O5—C30—C31	−0.4 (3)
C5—C4—C7—C13	171.5 (2)	C28—C29—C30—O5	−178.3 (2)
O1—C5—C8—C9	−64.4 (2)	C28—C29—C30—C31	1.4 (3)
C12—C5—C8—C9	53.7 (2)	O5—C30—C31—C26	178.2 (2)
C4—C5—C8—C9	−176.92 (18)	C29—C30—C31—C26	−1.5 (3)
C5—C8—C9—C10	−55.5 (3)	C27—C26—C31—C30	1.0 (3)
C8—C9—C10—C11	56.0 (3)	C25—C26—C31—C30	179.3 (2)
C9—C10—C11—C12	−54.9 (3)	C28—O4—C32—C33	−178.27 (18)
O1—C5—C12—C11	65.4 (2)	O4—C32—C33—C34	−16.0 (3)
C4—C5—C12—C11	−179.65 (18)	O4—C32—C33—C38	165.3 (2)
C8—C5—C12—C11	−52.2 (2)	C38—C33—C34—C35	−0.9 (3)
C10—C11—C12—C5	53.1 (3)	C32—C33—C34—C35	−179.6 (2)
C7—C4—C14—C15	−8.8 (4)	C33—C34—C35—C36	0.3 (4)
C5—C4—C14—C15	169.2 (2)	C34—C35—C36—C37	0.0 (4)
C4—C14—C15—C16	176.8 (2)	C35—C36—C37—C38	0.4 (4)
C14—C15—C16—C17	174.1 (2)	C36—C37—C38—C33	−1.0 (4)
C14—C15—C16—C23	−3.2 (3)	C34—C33—C38—C37	1.3 (4)
C15—C16—C17—C21	−171.2 (2)	C32—C33—C38—C37	180.0 (2)
C23—C16—C17—C21	6.1 (3)	C30—O5—C39—C40	83.2 (3)
C15—C16—C17—C18	9.2 (3)	O5—C39—C40—C41	38.8 (3)
C23—C16—C17—C18	−173.5 (2)	O5—C39—C40—C45	−146.5 (2)
C21—C17—C18—C19	2.9 (3)	C45—C40—C41—C42	−0.2 (4)
C16—C17—C18—C19	−177.4 (2)	C39—C40—C41—C42	174.5 (2)
C20—N4—C19—C18	−1.5 (3)	C40—C41—C42—C43	−1.3 (4)
C22—N4—C19—C18	175.8 (2)	C41—C42—C43—C44	2.0 (4)
C17—C18—C19—N4	−1.0 (3)	C42—C43—C44—C45	−1.1 (4)
C19—N4—C20—C21	1.9 (3)	C43—C44—C45—C40	−0.5 (4)
C22—N4—C20—C21	−175.3 (2)	C41—C40—C45—C44	1.2 (4)
N4—C20—C21—C17	0.2 (3)	C39—C40—C45—C44	−173.6 (2)

### Hydrogen-bond geometry (Å, º)

Cg1 is the centroid of the C33–C38 phenyl ring.

**Table:**

*D*—H···*A*	*D*—H	H···*A*	*D*···*A*	*D*—H···*A*
C8—H8*A*···N3^i^	0.99	2.54	3.504 (3)	163
C19—H19···N2^ii^	0.95	2.47	3.347 (3)	153
C22—H22*B*···N2^ii^	0.98	2.59	3.454 (3)	147
C29—H29···O5^iii^	0.95	2.54	3.430 (3)	156
C12—H12*A*···*Cg*1^iii^	0.99	2.55	3.542 (3)	177

Symmetry codes: (i) −*x*+1/2, −*y*+3/2, −*z*+1; (ii) −*x*+1/2, *y*+1/2, −*z*+3/2; (iii) −*x*+1, −*y*+2, −*z*.
